# Uncovering the Beneficial Role of *Limosilactobacillus fermentum* E7 Exhibiting Antioxidant Activity in Ameliorating DSS-Induced Ulcerative Colitis in a Murine Model

**DOI:** 10.3390/foods14010137

**Published:** 2025-01-06

**Authors:** Hongyan Zhang, Hailing Wang, Yue Li, Yue Leng, Ke Lin, Dayong Ren

**Affiliations:** College of Food Science and Engineering, Jilin Agricultural University, Changchun 130118, China; zhanghongyan_1016@163.com (H.Z.); wang_152326@163.com (H.W.); liyue_213@126.com (Y.L.); lengyue@jlau.edu.cn (Y.L.); rendayong@jlau.edu.cn (D.R.)

**Keywords:** hyperuricemia, *Limosilactobacillus fermentum* E7, antioxidant activity, ulcerative colitis, intestinal barrier function, gut microbiota

## Abstract

Background: Ulcerative colitis (UC) is a chronic intestinal disease of growing global concern. Bacteria associated with fermented food or probiotics regulate immune and inflammatory responses, playing a key role in intestinal immune homeostasis. Results: Five probiotics with relatively good antioxidant effects, namely *Lactiplantibacillus plantarum* H6, *Latilactobacillus sakei* QC9, *Limosilactobacillus fermentum* E7, *Bacillus subtills* D1, and *Bacillus licheniformis* Q13, were screened out from 30 strains of probiotics through in vitro antioxidant assays. The five probiotics had varying degrees of alleviating effects on UC mice and improved various physiological indicators, such as oxidative stress parameters and histopathological sections. The effects of E7, D1, and Q13 were more pronounced. Furthermore, E7 effectively regulated UC mouse intestinal microbiota composition, increased short-chain fatty acid concentration, and promoted the expression of anti-inflammatory factors, such as interleukin 10 (IL-10), while suppressing that of pro-inflammatory factors, such as interleukin 1β (IL-1β), interleukin 6 (IL-6), and tumor necrosis factor α (TNF-α). Meanwhile, D1 and Q13 only exhibited partial alleviating effects. Finally, E7 increased the expression of tight junction proteins in colon tissues. Conclusions: E7 showed superior efficacy to other probiotics in alleviating UC, offering novel therapeutic prospects for safer and effective management of UC.

## 1. Introduction

Ulcerative colitis (UC) is a complex gastrointestinal disorder caused by genetic susceptibility and a maladaptive immune response. Drugs commonly used to treat colitis include mesalazine [1], immunosuppressants [2], biological agents [3], small molecular agents [3], and methylprednisolone [4]. However, although these drugs are more effective than placebos in alleviating the symptoms of colitis, some patients do not respond to them or experience severe side effects, such as herpes zoster, cardiovascular diseases, and tumors [5]. Thus, novel methods for treating UC or restricting its side effects must be developed.

Currently, it is widely accepted that the pathogenesis of UC is associated with oxidative stress imbalance, intestinal mucosal damage, alterations in the composition of the gut microbiota, abnormal immune responses, and potential genetic and environmental factors [6,7]. Research has shown that maintaining oxidative stress homeostasis within the human gut plays a crucial role in the onset and progression of UC [8]. Oxidative stress (OS) refers to a state in which there is an imbalance between oxidation and anti-oxidation in the body, which tends to cause an increase in the presence of oxidized reactive oxygen species (ROS) in internal and external environments through harmful stimulation, leading to physiological and pathological damage to cells and tissues [7]. Moreover, OS can activate the inflammatory corpuscle complex of NOD-like receptor thermos protein domain-related protein 3 (NLRP3), promote the production of inflammatory pyrophosphate, and finally destroy the colonic mucosal structure, inducing UC [9].

Numerous studies have demonstrated a positive correlation between the dysregulation of the human gut environment and the onset and progression of UC [10]. UC patients often exhibit dysregulation of the intestinal immune system and imbalance of the gut microbiota [11]. A notable manifestation is the impairment of intestinal epithelial integrity, which is essential for maintaining intestinal homeostasis. Observations indicate that in patients with UC, the intestinal epithelial mucosa undergoes shedding, crypt and goblet cell loss, and inflammatory cell infiltration before ultimately forming ulcers [12]. In addition, the intestinal mucosa is covered in a thick mucosal layer, enabling intestinal bacteria to colonize and create a unique immune environment [13]. Specific probiotics that can breakdown mucus, such as *Akkermansia muciniphila* and *Bacteroides fragilis*, play a crucial role in colitis prevention by utilizing the outer mucosal layer [14]. Goblet cells are a specific type of epithelial cells that are essential to the production and secretion of mucus and maintain the integrity of the internal mucosal layer [15]. When goblet cells and tight junction proteins are abnormally expressed, the immune system can be compromised and becomes susceptible to harmful bacteria and toxins [16]. The epithelial barrier serves as the primary line of defense against such harmful substances. Damage to this barrier can increase the colon’s permeability and exacerbate the symptoms of colitis [17].

The health of the intestines relies heavily on the homeostasis of the gut microbiota. Disruptions in this balance can enable opportunistic pathogens to colonize and invade the intestines, triggering immune responses in the host and the onset of UC [18]. In UC patients, a decrease in *Eubacterium rectale* and *Akkermansia muciniphila* and an increase in *Escherichia coli* has been found [19]. Short-chain fatty acids (SCFAs) are the final products of microbial metabolism in the intestine and are considered key mediators between gut microbes and the host immune system [20]. SCFAs regulate the immunity of the intestinal mucosa by promoting the proliferation of B cells and the generation and expansion of regulatory T cells (Tregs). Butyric acid can influence immune cells in the gut lining, improving Treg function and subsequently inhibiting the activity of neutrophils, macrophages, and dendritic cells. The imbalance of the gut microbiota and increase in inflammatory cells in individuals with UC have been associated with decreased SCFA levels [21]. A decline in the population of bacteria that produce butyric acid in the gut (such as *Clostridium butyricum*) has been associated with a reduction in SCFA levels [22]. Consequently, probiotic supplementation based on microbial therapy is a novel method for alleviating UC or eradicating the adverse reactions induced by medications.

Supplementation by probiotics, which are the most abundant bacteria in fermented food, has emerged as a novel approach for alleviating gastrointestinal disorders [23]. Owing to increasing understanding of the function of the intestinal microflora, various probiotics, including lactic acid bacteria and *Bacillus*, have been found to be beneficial for human health. These beneficial microorganisms can colonize the intestinal tract and enhance the population of beneficial bacteria while reducing the diversity of detrimental bacteria [24]. It is universally known that *Bifidobacterium* and *Lactobacillus*, which are the two principal categories of probiotics, can competitively eradicate harmful microorganisms, producing antibacterial substances, regulating immune function, and restoring the symbiotic relationship within the intestines [25]. Furthermore, their capacity to produce copious amounts of SCFAs contributes to the alleviation of inflammatory bowel disease (IBD) [26]. Research has uncovered that the utilization of *Bacillus subtilis* can facilitate recovery from an intestinal mucosal injury resulting from DSS in mice. *Lactobacillus acidophilus* 1 prevents the development of murine ulcerative colitis by enhancing intestinal epithelial tight junction expression [27]. However, studies have shown that certain probiotics exhibit strain-specific effects. For instance, compared to *Akkermansia muciniphila* strain 193, ATCC significantly reduced spleen weight, colitis index, and fecal lipopolysaccharide-2 levels in mice with colitis [28]. The inter-strain differences in the improvement of IBD by 120 strains of *Lactobacillus sakei* have also been examined. The results showed that, compared to strain QJSNT1L10, strain CCFM1276 was effective in increasing the concentration of SCFAs, thereby alleviating IBD in mice [29]. Therefore, the effective utilization of probiotics is a crucial strategy for combating and treating colitis. However, due to the variability in UC improvement among different species and even among different strains of the same species, specific research on particular strains is necessary.

In this study, we successfully isolated 30 probiotics from traditional fermented food, aiming to select beneficial bacteria that can effectively improve UC through in vitro antioxidant and in vivo colitis mouse model experiments. Due to the widespread use of mouse models in basic research and functional evaluation, we established a UC mouse model by adding DSS to their drinking water. By screening probiotics through physical and chemical indicators, gut microbiota, SCFAs, and inflammatory factors, the most effective probiotic in alleviating colitis was identified. This could potentially result in a safe, cost-effective, and easy-to-administer method for the prevention or treatment of UC.

## 2. Materials and Methods

### 2.1. Antioxidant Ability of Probiotics In Vitro

#### 2.1.1. Bacterial Strains and Preparation

Probiotics were cultured for 12–48 h at 37 °C. Then, the bacterial culture was centrifuged at a speed of 4000× *g* at a temperature of 4 °C for 10 min. The bacterial precipitate was washed twice with phosphate buffer solution (PBS; pH 7.2) to remove impurities. The bacterial suspension was prepared by adjusting the final bacterial concentration to 1.0 × 10^9^ CFU/mL using PBS. Table S1 contains the names of the bacterial strains.

#### 2.1.2. DPPH Radical Scavenging Capacity

A measure of 1 mL of bacterial suspension (1.0 × 10⁹ CFU/mL) was mixed with 1 mL of 0.2 mmol/L DPPH ethanol solution and incubated at 37 °C in the dark for 30 min. The bacterial solution was substituted with an equal volume of PBS, and DPPH absolute ethanol solution served as the blank group. The solution was then cultured under the same conditions. Centrifugation was conducted at a speed of 7000× *g* for 10 min, and the absorbance of the supernatant was measured using a multifunctional enzyme label instrument at a wavelength of 517 nm. The performance of probiotics in inhibiting DPPH free radical was evaluated using the following formula.

$$\mathrm{DPPH}\;\mathrm{free}\;\mathrm{radical}\;\mathrm{scavenging}\;\mathrm{rate}\;(\%)=(1-\frac{{A517}_{sample}}{{A517}_{blank}})\times 100\%.$$

#### 2.1.3. ABTS Radical Scavenging Capacity

The working solution of ABTS contained 7.4 mmol/L ABTS solution and 2.6 mmol/L potassium persulfate solution at a ratio of 1:1 (*v*/*v*). This was incubated in the dark for 12–24 h at room temperature and diluted with PBS (0.1 mmol/L, pH 7.2) so that the absorbance at 734 nm was 0.7 ± 0.02. Each measurement was conducted by mixing 200 μL of the bacterial suspension with 1.0 × 10^9^ CFU/mL and 0.8 mL of the ABTS working solution. After 6 min at 30 °C, absorbance at 734 nm was measured with a blank, using distilled water instead of the bacterial suspension. The following formula can be used to calculate the free radical scavenging ability of ABTS.

$$\mathrm{ABTS}\;\mathrm{radical}\;\mathrm{scavenging}\;\mathrm{capacity}\;(\%)=\frac{{A734}_{blank}-{A734}_{sample}}{{A734}_{sample}}\times 100\%.$$

#### 2.1.4. Hydroxyl Radical Scavenging Activity

First, 1 mL of 1,10-phenanthroline, 1 mL of PBS (pH 7.2), 1 mL of bacterial suspension (1.0 × 10^9^ CFU/mL), and 1 mL of FeSO_4_ were mixed in a test tube. Then, 1 mL of 20 mmol/L H_2_O_2_ was added, and the mixture was heated in a water bath at 37 °C for 1.5 h. The absorbance was measured at 536 nm. The resulting solution represented the experimental group. Then, PBS with the same volume as the blank group was used to replace the bacterial suspension. Finally, distilled water was used as the control group, replacing H_2_O_2_ under the same culture conditions and detection methods. The performance of probiotics in scavenging hydroxyl radicals was assessed using the following formula.

$$\mathrm{Hydroxyl}\;\mathrm{radical}\;\mathrm{scavenging}\;\mathrm{rate}\;(\%)=\frac{{A536}_{sample}-{A536}_{blank}}{{A536}_{control}-{A536}_{blank}}\times 100\%.$$

#### 2.1.5. Reducing Ability Determination

The reducing power of probiotics was assessed by mixing 0.5 mL of a bacterial suspension (1.0 × 10^9^ CFU/mL), 0.5 mL of 1% potassium ferricyanide, and 0.5 mL of PBS buffer (pH 6.6) and shaking vigorously. The control group consisted of distilled water, potassium ferricyanide, and PBS buffer. The reaction occurred at 50 °C for 20 min, followed by rapid cooling and the addition of 0.5 mL of 10% trichloroacetic acid. After centrifugation at 2000× *g* for 5 min, 1 mL of the supernatant was mixed with 1 mL of 0.1% ferric chloride, and the mixture was allowed to stand in the dark at room temperature for 10 min. Absorbance was then measured at 700 nm. Finally, different concentrations of cysteine were prepared via a reaction with distilled water instead of the bacterial suspension, and the reducing power was measured according to its absorbance.

### 2.2. Animal Experiments

In this study, C57BL/6J male mice aged 6–8 weeks were sourced from Beijing Huafukang Biotechnology Co. Ltd. They were kept in a controlled environment at 25 ± 2 °C and 50% ± 5% humidity, with a consistent 12 h light/dark cycle. All animal experimental procedures were conducted in accordance with the “Guidelines for the Care and Use of Laboratory Animals at Jilin Agricultural University” and were approved by the Animal Ethics Committee of Jilin Agricultural University (SYXK [JI] 2018-0023).

During the 14-day study, the animals were divided into several groups: control, DSS, and five probiotic groups treated with different bacteria (*L. plantarum* H6, *L. sakei* QC9, *L. fermentum* E7, *B. subtilis* D1, *B. licheniformis* Q13). Initially, for the first seven days, all groups except the control group were given 3.5% DSS to induce colitis. Following this, all groups had access to free drinking water from day eight. The control and DSS groups received PBS, while the other groups received their respective bacterial treatments at 1 × 10^9^ CFU/kg/day.

### 2.3. Measurement of Disease Activity Index (DAI)

During the experiment, daily measurements were taken of body weight, food intake, fecal consistency, and occult blood, and the disease activity index (DAI) score was calculated for each mouse. Furthermore, the DAI for each mouse was determined using the following equation: DAI = sum of scores for weight loss, food consumption, and combined scores for stool consistency and bleeding. Table S2 delineates the grading criteria used for the DAI scores.

### 2.4. Histological Analysis

Colon tissues were fixed in 4% paraformaldehyde, stained with hematoxylin and eosin, dehydrated, embedded in paraffin, and sectioned into 4 mm-thick slices. The sections were observed under a microscope, and photographs were taken to detail the tissue structure.

### 2.5. Immunofluorescence (IF) Staining

After dewaxing, the tissue sections were submerged in a citric acid buffer (pH 6) for antigen repair. Subsequently, tissue sections were sealed by incubating them with 10% goat serum at 37 °C for 30 min. The glass slides were rinsed and incubated at 37 °C for 2 h with specific antibodies: anti-ZO-1, anti-Occludin, anti-claudin-1 (all PTG, 1:200), and anti-mucin-2 (Servicebio, 1:200). A secondary antibody was prepared using Tris-buffered saline with Tween 20 (TBST) at a dilution of 1:100 and incubated at 37 °C for 1 h. DAPI was used for nuclear staining, and KF-Viewer software (ver:1.7.0.27) was utilized for observation and analysis.

### 2.6. Western Blot Analysis

Colon tissue was homogenized (n = 3), and proteins were extracted using a total protein extraction kit (BestBio #BB-3101-100T, Shanghai, China). The BCA protein assay (Beyotime #P0012, Shanghai, China) was utilized to determine protein concentrations. The samples were then subjected to a heat treatment in a water bath at 100 °C for 10 min and subsequently analyzed using SDS agarose gel electrophoresis. The resolved proteins were transferred to a PVDF membrane, which was subsequently washed with TBST for a total of 15 min. The membrane was incubated with 5% BSA at room temperature for one hour. After washing with TBST, it was incubated overnight with primary antibodies (rabbit anti-claudin-1, anti-occludin, and anti-β-actin from Affinity at dilutions of 1:3000, 1:3000, and 1:20,000, respectively) at 4 °C. Following another TBST wash, the membrane was incubated with horseradish peroxidase-linked secondary antibody for 1.5 h at room temperature. Finally, the target proteins were detected using a chemiluminescence system (Image Quant LAS500 gel imaging system) and quantified by ImageJ software (1.52i).

### 2.7. Detection of Oxidative Stress Index and Inflammatory Factors in Colonic Tissue

The activities of myeloperoxidease (MPO), malondialdehyde (MDA), superoxide dismutase (SOD), and glutathione peroxidase (GSH-Px) in the colon tissues of each group were detected using a kit provided by Nanjing Jiancheng Technology Co., Ltd. (Nanjing, China). The ELISA kit was utilized to quantify the protein expression levels of IL-1β, IL-6, TNF-α, and IL-10 in the colon tissues following the manufacturer’s guidelines. Five samples were tested from each group.

### 2.8. Quantitative RT-PCR

RNA was isolated from colon tissues using a TriZol kit (Sangon Biotech, Shanghai, China, Thermo Fisher Scientific) as per the guidelines given by the manufacturer. Subsequently, this RNA was converted into cDNA using a Fast King RT Supermix solution (#KR118-02, Tiangen, Beijing, China). A real-time PCR reaction system was established with a Super Real PreMix Plus (SYBR Green) kit (#FP205, Tiangen, Beijing, China), and a two-step PCR amplification reaction was carried out. The conditions of amplification reaction included a predenaturation step at 95 °C for 15 min, a cycling step at 95 °C for 10 s, a cycling step for 40 repeats, and a final annealing step at 60 °C for 30 s. The relative expression of genes was determined by applying the 2^−∆∆Ct^ method. The target gene sequence is shown in Table S3. Three samples were tested from each group.

### 2.9. RNA Extraction, 16S rRNA Amplicon Sequencing, and Data Analysis

DNA in the feces of mice was sequenced using 16sRNA technology (eight samples from each group). Next, Omega Bio-tel (Norcross, GA, USA) was used to extract DNA and measure its concentration and purity. The V3-V4 hypervariable region of the 16sRNA gene of bacteria was amplified by PCR with specific primers, and the strains were classified. The PCR products were then quantitatively analyzed using Bio Tek (FLx 800, Seattle, WC, USA) and a Quant-iT Pico Green dsDNA detection kit (Invitrogen, Carlsbad, CA, USA). After purification, sequencing and library construction were completed using the Illumina Nova Seq platform (San Diego, CA, USA). Lastly, the bioinformatics analysis was carried out using QIIME2 2019.4.

### 2.10. SCFA Concentration Analysis

Cecum contents (50 mg) were placed in a centrifuge tube, and 0.5 mL of normal saline and steel balls were added (eight samples from each group). The solution was vortexed for 15 min and then centrifuged at 12,000× *g* at 4 degrees for 10 min. Then, the supernatant was collected and analyzed by GC-MS. Gas chromatography was conducted using a Trace 1310 gas chromatograph (Thermo Fisher Scientific Company, Waltham, MA, USA), with helium serving as the carrier gas. The flow rate was maintained at 1.2 mL/min, and the injection volume was 1 microliter. The chromatographic column temperature increased gradually from the initial 90 °C to 120 °C, then to 150 °C, and finally to 250 °C at a rate of 10, 5, and 25 °C/min, respectively, over 2 min. An LT ISQ mass spectrometer (Thermo Fisher Science, USA) was used for the identification of metabolites in electron impact ionization mode.

### 2.11. Statistical Analysis

GraphPad Prism 9.5.1 software (GraphPad Software Company, San Diego, CA, USA) was utilized for statistical analysis. The statistical significance was assessed using both one-way and two-way ANOVA, and the results were expressed as the average with standard deviation. A *p* value of less than 0.05 was considered statistically significant. Each experiment consisted of multiple samples. The sample count is shown in the figure legend. Representative images of the protein blots were derived from a minimum of three independent samples. Microbial data were analyzed using the R programming language. Principal component analysis (PCA) and orthogonal partial least squares discriminant analysis (OPLS-DA) are commonly employed in metabonomics to reduce the dimensionality of the sample data.

## 3. Results

### 3.1. Screening of Antioxidant Probiotics In Vitro

In our laboratory, 30 probiotic strains were isolated from traditional fermented food (Supplementary Table S1). Antioxidant assays were performed on the bacterial suspensions of the strains (n = 30; Table 1). A total of 26 strains demonstrated DPPH free radical scavenging activity, 29 strains showed hydroxyl free radical scavenging activity, and all strains demonstrated ABTS free radical scavenging activity and reducing ability. The scavenging rates of DPPH, ABTS, and hydroxyl radicals were 10.50% ± 1.17%–37.63% ± 2.22%, 7.62% ± 2.78%–38.37% ± 2.72%, and 12.23% ± 0.90%–60.15% ± 2.44%, respectively. The reducing ability was between 77.70 ± 5.27 and 289.80 ± 3.48 μmol/L. Strains H6, QC9, E7, D1, and Q13 demonstrated good antioxidant capacity based on the results of the four in vitro antioxidant tests. Therefore, these five strains of probiotics were tested in vivo.

### 3.2. Probiotics Substantially Reduce the Symptoms of Ulcerative Colitis in Mice

**Figure 1:**
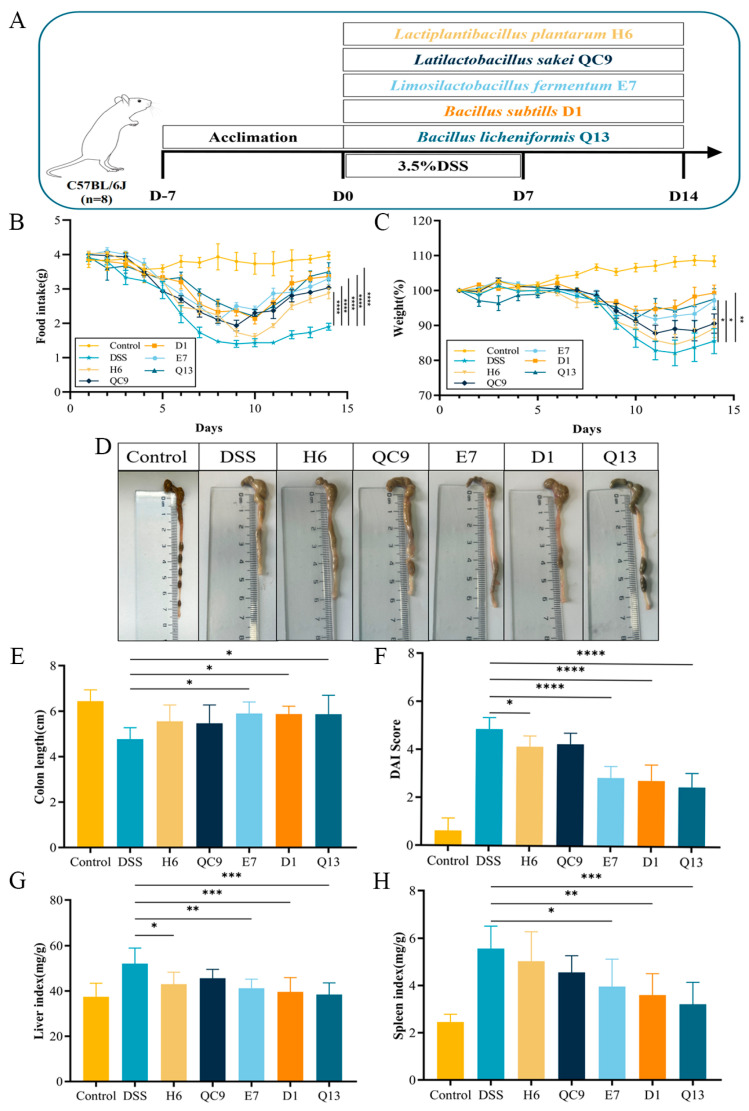
Probiotic intervention improved the physiological indicators of colitis in mice. (**A**) Grouping and experimental approach. (**B**) Food intake. (**C**) Weight loss. (**D**,**E**) Colon length. (**F**) DAI score. (**G**,**H**) Liver and spleen indices, respectively. Control, normal control group; DSS, DSS-induced model group; H6, *Lactiplantibacillus plantarum* H6 group; QC9, *Latilactobacillus sakei* QC9 group; E7, *Limosilactobacillus fermentum* E7 group; D1, *Bacillus subtills* D1 group; Q13, *Bacillus licheniformis* Q13 group. Data are mean ± SD of eight independent samples. * *p* < 0.05; ** *p* < 0.01; *** *p* < 0.001; **** *p* < 0.0001.

To study the effect of probiotics from traditional fermented food on UC, we induced a colitis model in mice by adding 3.5% DSS to their drinking water (Figure 1A). The measured food intake, body weight, DAI score, colon length, and organ index values revealed a preliminary relieving effect of probiotics on UC in mice. Figure 1B shows that all five probiotics considerably increased food intake in mice compared to the DSS group. In addition, the probiotics E7, D1, and Q13 inhibited weight loss in mice, reduced their DAI scores, and decreased their immune organ index scores (Figure 1C–H). The H6 and QC9 groups showed some improvements in these indicators, but the results were mostly not significant.

### 3.3. Intervention of Probiotics Alleviated Oxidative Stress in Colitis Mice

**Figure 2:**
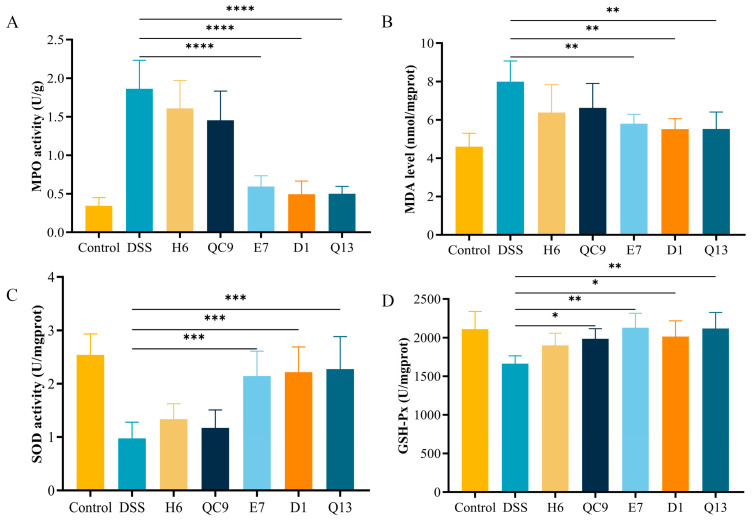
Effect of probiotics on oxidative stress in the colon of mice. (**A**) MPO activity, (**B**) MDA level, (**C**) SOD activity, (**D**) GSH-Px activity. Control, normal control group; DSS, DSS-induced model group; H6, *Lactiplantibacillus plantarum* H6 group; QC9, *Latilactobacillus sakei* QC9 group; E7, *Limosilactobacillus fermentum* E7 group; D1, *Bacillus subtills* D1 group; Q13, *Bacillus licheniformis* Q13 group. Data are mean ± SD of eight independent samples. * *p* < 0.05; ** *p* < 0.01; *** *p* < 0.001; **** *p* < 0.0001.

OS is the key factor affecting UC progression, which mainly manifests as a result of an imbalance between the production and elimination of ROS. Therefore, the repair effects of five probiotics on oxidative imbalance in DSS-treated mice were evaluated. After DSS treatment, the MPO activity and MDA level in the colons of mice increased significantly, indicating that the level of OS in mice increased. The MPO activity and MDA level in mice were attenuated after probiotic intervention (Figure 2A,B). In addition, SOD activity and GSH-Px activity in the DSS group decreased significantly, but this effect was mitigated by probiotic intervention (Figure 2C,D).

### 3.4. Probiotics Mitigated Colon Tissue Damage

**Figure 3:**
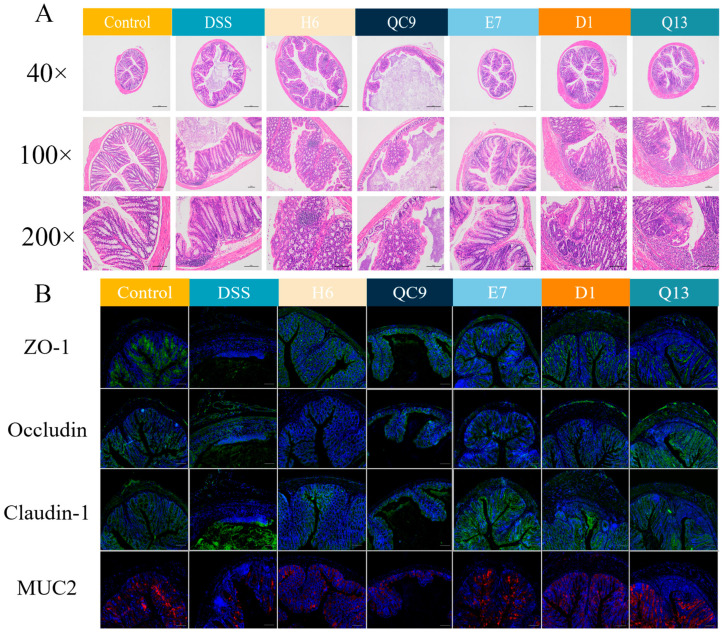
Probiotics mitigated colon tissue damage. (**A**) Hematoxylin and eosin (H&E) staining of mouse colonic tissues at magnifications of 40×, 100×, and 200×. The scales of the three models are 500, 200, and 100 μm, respectively. (**B**) Immunofluorescent staining of three closely associated proteins (ZO-1, occluding, and claudin-1; green fluorescence) and mucin-2 (red fluorescence); scale bars, 100 μm. Control, normal control group; DSS, DSS-induced model group; H6, *Lactiplantibacillus plantarum* H6 group; QC9, *Latilactobacillus sakei* QC9 group; E7, *Limosilactobacillus fermentum* E7 group; D1, *Bacillus subtills* D1 group; Q13, *Bacillus licheniformis* Q13 group. Data are mean ± SD of three independent samples.

The colon tissue was stained with H&E stain to explore the effects of five probiotics on UC in mice. Compared with the control group, the DSS group showed serious pathological changes, including extensive crypt injury, an obvious decrease in goblet cells, and a large influx of inflammatory cells. Colon tissue loss in the H6 and QC9 groups was severe, similar to the colon tissues in the DSS group. Despite some inflammatory cell aggregation, the results obtained from the D1 and Q13 groups were essentially normal, with less crypt and goblet cell loss, indicating the overall integrity of the colonic tissue. The E7 group showed significantly improved symptoms, demonstrating preserved crypt architecture, abundant goblet cells, and limited inflammatory infiltration (Figure 3A).

We examined the expression of three TJ proteins (ZO-1, occludin, and claudin-1) and mucin-2 in colon tissues via IF staining. Compared to the DSS group, the fluorescence intensity of TJ protein and MUC2 increased in the E7, D1, and Q13 groups. However, the fluorescence expression intensities in the H6 and QC9 groups were relatively weak (Figure 3B). The results indicate that E7, D1, and Q13 can alleviate the physiological and pathological damage induced by DSS in mouse colitis. Therefore, we further analyzed in subsequent experiments how these three probiotics alleviate UC.

### 3.5. Probiotics Can Regulate Gut Microbiota Composition in Mice with Colitis

**Figure 4:**
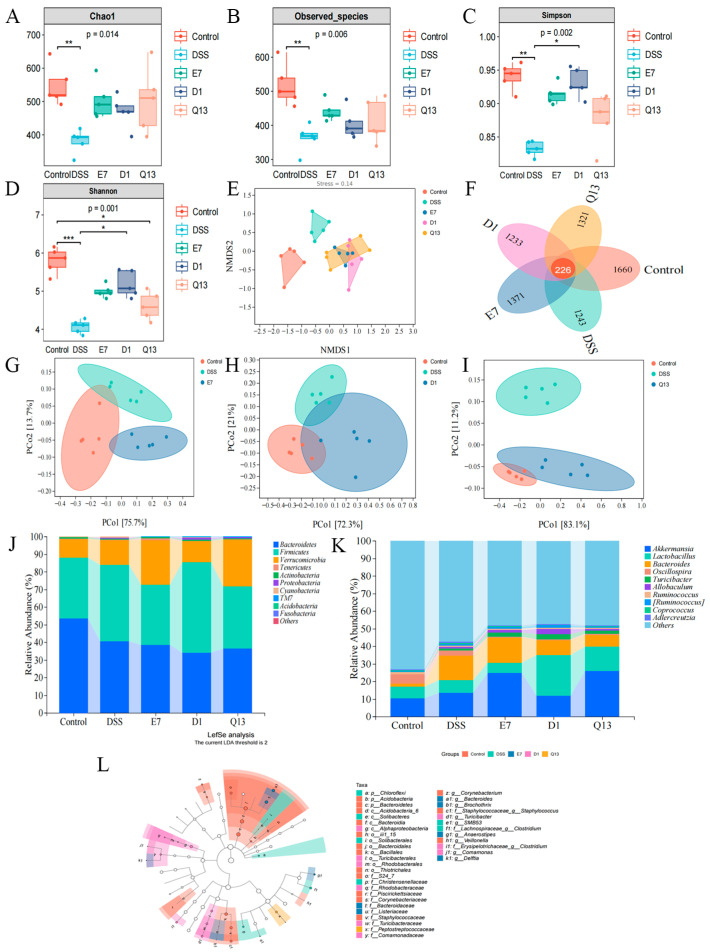
Probiotics regulate the composition of the gut microbiome in mice with colitis. (**A**) Chao1 index. (**B**) Observed_species index. (**C**) Simpson index. (**D**) Shannon index. (**E**) Non-metric multidimensional scaling (NMDS) analysis. (**F**) Venn diagram. (**G**–**I**) The principal coordinate analysis (PCoA) for the E7, D1, and Q13 groups, respectively. (**J**,**K**) The taxonomic composition at the phylum and genus levels for each group, respectively. (**L**) LefSe analysis. Control, normal control group; DSS, DSS-induced model group; E7, *Limosilactobacillus fermentum* E7 group; D1, *Bacillus subtills* D1 group; Q13, *Bacillus licheniformis* Q13 group. Data are mean ± SD of five independent samples. * *p* < 0.05; ** *p* < 0.01; *** *p* < 0.001.

To evaluate whether probiotics can alleviate UC in mice by regulating intestinal microflora composition, feces from each group were collected and analyzed by 16S rRNA sequencing. α diversity reflects the diversity and richness of intestinal microflora. Compared with the control group, the microbial diversity of the DSS group was significantly lower. However, this trend was reversed after intervention with probiotics (Figure 4A–D). The results of NMDS analysis and species difference Venn diagrams showed that the DSS group had a different microbial composition from the E7, D1, and Q13 groups (Figure 4E,F). β-diversity in different treatment groups was evaluated using principal coordinate analysis (PCoA), and the similarities between bacterial communities in these groups was elucidated (Figure 4G–I). The results showed that probiotic intervention changed the diversity and structural composition of the gut microbiota in mice with UC.

Subsequently, to further elucidate bacterial community characteristics within each group, we evaluated the effects of E7, D1, and Q13 on gastrointestinal microbiome composition and relative abundance. At the level of phylum, compared with the DSS group, the abundance of Verrucomicrobia in the E7 and Q13 groups increased significantly, and the abundance of Firmicutes in the D1 group increased significantly. In addition, the abundance of Bacteroidetes decreased significantly after treatment with the three probiotics (Figure 4J). At the genus level, the abundances of *Akkermansia*, *Turicibacter*, and *Allbaculum* significantly increased in the E7 group. The abundances of *Lactobacillus*, *Turicibacter*, *Allbaculum*, and *Rminococcus* significantly increased in the D1 group. The abundances of *Akkermansia*, *Lactobacillus*, and *Turicibacter* significantly increased in the Q13 group (Figure 4K). LefSe analysis further clarified the differences in species among the groups (Figure 4L). These results indicated that probiotics can alleviate UC in mice by adjusting the composition of intestinal microflora.

### 3.6. Probiotics Increased the Content of SCFAs in Colitis Mice

**Figure 5:**
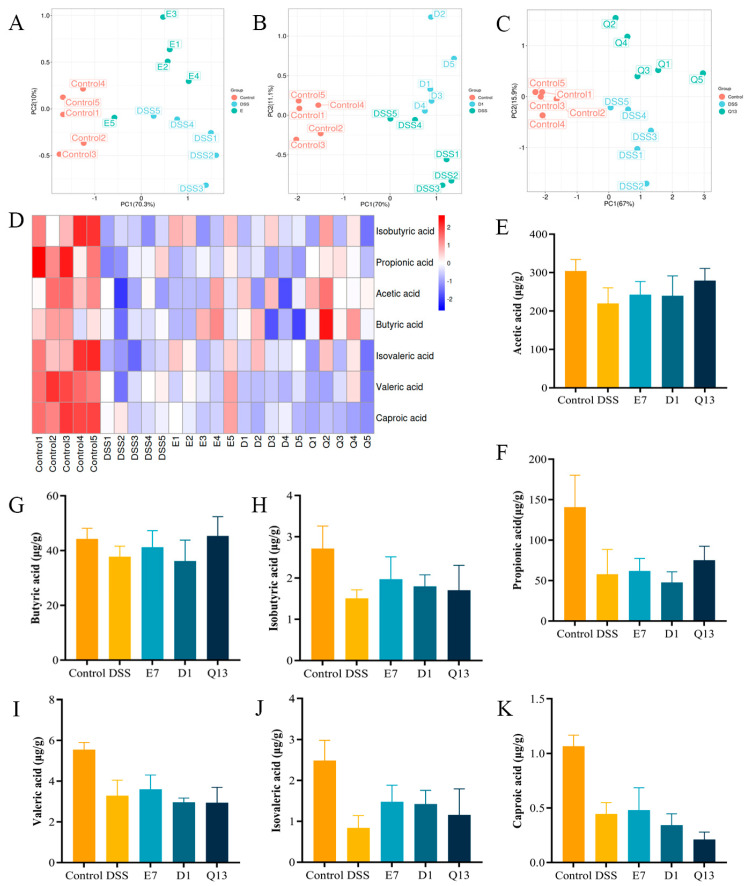
Effect of probiotics on the SCFAs in the cecal content of colitis mice. (**A**–**C**) Partial least square discriminant analysis (PLS-DA) for E7, D1, and Q13, respectively. (**D**) Overall metabolome clustering heat map. (**E**) Acetic acid; (**F**) Propionic acid; (**G**) Butyric acid; (**H**) Isobutyric acid; (**I**) Valeric acid; (**J**) Isovaleric acid; and (**K**) Caproic acid. Control, normal control group; DSS, DSS-induced model group; E7, *Limosilactobacillus fermentum* E7 group; D1, *Bacillus subtills* D1 group; Q13, *Bacillus licheniformis* Q13 group. Data are mean ± SD of five independent samples.

SCFAs are the final products of gut microbiota metabolism and play an important role in interactions between the microbiome and immune system. Gut microbiome composition and abundance consistently influence microbial metabolites. Therefore, we measured the concentrations of SCFAs in the cacum of each group through GC–MS. As shown in Figure 5A–C, dimensional reduction analysis of SCFAs in the E7, D1, and Q13 groups indicated compositional differences between the DSS group and all three probiotic groups. Cluster thermal analysis showed that the content of SCFAs in the DSS group was signify inferior to the control group (Figure 5D). Quantitative analysis showed increased SCFAs after probiotic intervention, but the results were not significant (Figure 5E–K). The results indicate that treatment with beneficial bacteria can regulate the concentrations of SCFAs in the cecum content of colitis mice.

### 3.7. Probiotics Improved Inflammatory Factors in Colonic Tissues and Increased the Expression of TJ Proteins

**Figure 6:**
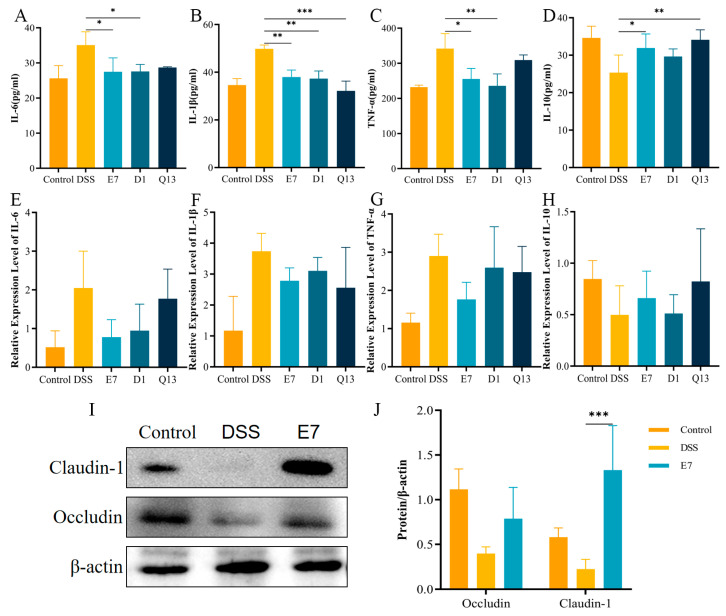
Probiotics improved inflammatory factors in colon tissues. (**A**–**D**) The protein levels of IL-6, IL-1β, TNF-α, and IL-10 in the colon tissues were detected using an ELISA kit (Shanghai, China). (**E**–**H**) The gene levels of IL-6, IL-1β, TNF-α, and IL-10 were detected in the colorectal tissues using RT-qPCR. (**I**) Western blot analysis of occludin and claudin-1. (**J**) Quantitative analysis of the Western blotting of occludin and claudin-1. Control, normal control group; DSS, DSS-induced model group; E7, *Limosilactobacillus fermentum* E7 group; D1, *Bacillus subtills* D1 group; Q13, *Bacillus licheniformis* Q13 group. Data are mean ± SD of three independent samples. * *p* < 0.05; ** *p* < 0.01; *** *p* < 0.001.

The oversecretion of inflammatory cytokines is closely related to IBD and its clinical symptoms in the intestine. We used RT-qPCR and ELISA kits to evaluate inflammatory cytokines in colon tissues. Specifically, we measured the expression levels of IL-6, IL-1β, TNF-α, and IL-10. The protein levels showed that the expression level of pro-inflammatory factors in E7 group was significantly lower than that in DSS group, while the expression level of anti-inflammatory factors was higher. The levels of the three proinflammatory factors in the D1 group significantly decreased. The Q13 group showed significant reduction in IL-1β level and increase in IL-10 expression level (Figure 6A–D). Figure 6E–H shows that the expression levels of the pro-inflammatory factors significantly increased, whereas those of the anti-inflammatory factors significantly decreased in the DSS group compared with the control group at the gene level. After the administration of the three probiotics into the stomach, a shift in trend was observed, although it was not statistically significant. The study indicates that probiotics can improve UC by adjusting colonic inflammatory factors, with the E7 group showing the most significant improvement.

Therefore, we detected the reflection levels of claudin-1 and occludin in the E7 group by Western blot analysis to verify whether E7 can alleviate UC by improving colon tissue injury. As shown in Figure 6I,J, the expression level of TJ protein in the E7 group was higher than that in DSS group. This result further demonstrated that among the five probiotics, the fermentation of E7 better improved murine ulcerative colitis.

### 3.8. Correlations Among the Gut Microbiota, Metabolites, and Clinical Parameters

**Figure 7:**
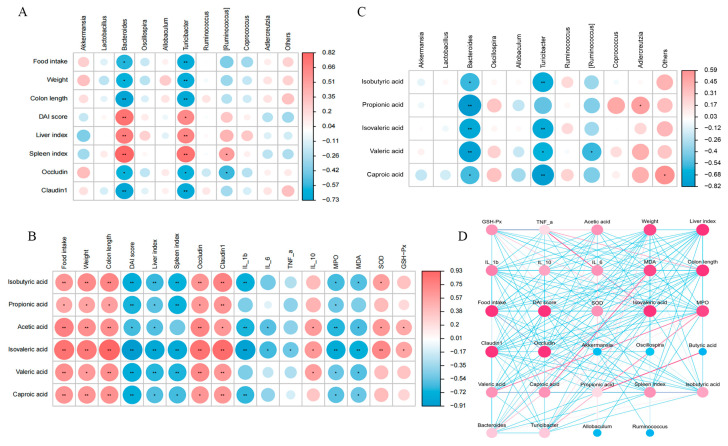
Correlations among the gut microbiota, metabolites, and clinical parameters. (**A**) Heatmap depicting the correlation between clinical indicators and gut microbiome. (**B**) Heatmap of the correlation between clinical indicators and SCFAs. (**C**) Heatmap of the correlation between gut microbiome and SCFAs. (**D**) Network analysis of the correlations among clinical indicators, gut microbiome, and SCFAs. Control, normal control group; DSS, DSS-induced model group; E7, *Limosilactobacillus fermentum* E7 group; D1, *Bacillus subtills* D1 group; Q13, *Bacillus licheniformis* Q13 group. Data are mean ± SD of five independent samples. * *p* < 0.05; ** *p* < 0.01.

We revealed correlations among clinical indicators, gut microbiota, and SCFAs through Pearson analysis. As demonstrated in Figure 7A, *Akkermansia* and *Adlercreutzia* positively correlated with food intake, body weight, colon length, and tight junction proteins and negatively correlated with DAI scores and organ indices. By contrast, *Bacteroidetes* and *Turicibacter* showed the opposite trends. Correlation analysis showed that SCFAs correlated positively with food intake, body weight, colon length, and tight junction protein, IL-10, SOD, and GXH-Px levels but negatively with DAI scores, spleen indices, and pro-inflammatory factor, MPO, and MDA levels (Figure 7B). The heatmap results of the correlation between gut microbiota and SCFAs indicate a negative correlation between *Bacteroidetes* and *Turicibacter* (Figure 7C). There is a correlation between clinical indicators, gut microbiota, and metabolites, which is visualized in the network map (Figure 7D).

## 4. Discussion

An increasing number of studies have evaluated the potential function of strains isolated from fermented foods as probiotics [30,31]. Probiotics reduce colitis symptoms by improving intestinal barrier function and regulating inflammatory factors and the intestinal microbiota [32]. However, the immunomodulatory functions and mechanisms of beneficial bacteria from various sources have yet to be confirmed. This study aimed to isolate beneficial bacteria that can alleviate UC in mice from 30 strains from different sources of probiotics, using in vitro antioxidant and in vivo colitis mouse models. The combined results of the in vitro and in vivo experiments indicated that E7 could better improve the physicochemical indicators, intestinal barrier, immune system, and gut microbiome of mice with colitis.

The imbalance between OS and antioxidation is a key factor influencing the occurrence and development of UC [33]. Therefore, we screened five probiotics (H6, QC9, E7, D1, and Q13) with good antioxidative effects from thirty strains of probiotics using four in vitro antioxidative methods. Experiments on the colitis mouse model indicated that these probiotics alleviated UC in the mice to varying degrees. E7, D1, and Q13 restored the mice’s feeding ability, body weight, colonic length, DAI score, and organ index, reducing pathological damage. The activities of MPO, MDA, SOD, and GSH-Px are important indicators for evaluating OS in vivo [34]. Imbalances in the production and elimination of ROS are the major contributors to colitis pathogenesis [34]. Elevated MPO activity closely correlates with neutrophil infiltration in colon tissues. Infiltrated neutrophils can be released into colonic epithelial cells in the form of neutrophil extracellular trap network structure using ROS, causing colonic crypt cysts [35]. MDA is the end product of lipid peroxidation and can disrupt cellular integrity through protein cross-linking and polymerization [20]. SOD and GSH-Px are important antioxidant enzymes, and the elevated activities imply considerable reduction in oxidative damage to the body [36]. After five-strain probiotic intervention, the colon tissue’s OS state improved, and the E7, D1, and Q13 groups showed notable changes. The experimental results suggested that the probiotic strains can balance ROS production and antioxidant systems in the body through their innate antioxidant ability.

A complete intestinal barrier is vital for the physiological functions of the body and disease prevention [29]. The mucosal layer and tight junction proteins are important mediators for maintaining the integrity of the intestinal epithelium barrier. When the intestinal barrier is damaged, pathogens and endotoxins can enter the lumen, resulting in intestinal inflammation [37]. Barnaba et al. [38] showed that intestinal homeostasis and distinguishing harmful pathogens from beneficial gut microbes rely heavily on the balance between Tregs and helper T cell 17. This regulation is closely related to the gut microbiome, because gut microorganisms, such as Firmicutes phylum, *Akkermansia* spp., and *Lactobacillus* spp., can influence Treg cell production and subsequently affect the integrity of the intestinal barrier [39,40]. Mao et al. [20] found that *Blautia producta* D4 can increase the expression of tight junction proteins (ZO-1, claudin-1, and occludin) in colonic tissues, alleviating colonic tissue damage. Our research findings indicate that E7, D1, and Q13 can alleviate murine UC by enhancing the expression of mucin-2 and TJ proteins in colonic tissue. Therefore, subsequent experiments were conducted on these three probiotics.

The gut microbiome and its metabolites, particularly SCFAs, play an important role in IBDs [41]. Compared with healthy individuals, patients with UC have significantly lower abundances of *Akkermansia* and *Lactobacillus*. Notably, *Akkermansia muciniphila* upregulates genes involved in maintaining the function of the intestinal barrier via the ADP–glucose-dependent ALPK1/TIFA pathway [42]. *Lactobacillus* acts as an intestinal symbiont, aiding in the maintenance of intestinal health and body homeostasis [43]. Additionally, SCFAs can effectively maintain gut health and immunological homeostasis in the body. These acids primarily exert their anti-inflammatory effects by inhibiting the activity of pro-inflammatory cytokines in the intestinal epithelium and suppressing the activation of the NF-κB signaling pathway in macrophages [26]. Shan et al. [44] observed that SCFAs can repair damaged intestinal mucosa by stimulating goblet cells to secrete mucus. In patients with colitis, the relative abundances of some gut bacteria that produce SCFAs, such as *Akkermansia*, are reduced. This reduction leads to a decrease in the concentrations of SCFAs in the gut and limits the energy supply to colon epithelial cells and local control of mucosal inflammation [28]. SCFAs can inhibit the growth of pathogenic microorganisms, including *Escherichia coli* and *Salmonella*, in the intestine, competing with them for colonization sites [45]. The abundance of butyrate-producing species (e.g., *Faecalibacterium prausnitzii* and *Roseburia hominis*) contributes to immunological homeostasis in patients with UC [46,47]. Our study has found that among the three probiotics, E7 can better increase the concentration of certain bacteria that produce SCFAs (such as *Akkermansia*), thereby enhancing the expression of SCFAs.

Immunocytes produce cytokines that regulate inflammation, either promoting or inhibiting it. This can affect the development and progression of UC [48]. Pro-inflammatory cytokines cause intestinal inflammation and tissue damage and perpetuate diseases in UC while suppressing its resolution [49]. In patients with UC, immune dysregulation promotes the secretion of pro-inflammatory cytokines, such as TNF-α, IL-6, and IL-1β, enhancing lymphocyte activation and proliferation [50]. By contrast, anti-inflammatory cytokines maintain intestinal barrier integrity by stimulating cell proliferation, enhancing tight junction proteins, and repressing cell apoptosis [37]. For instance, IL-10 produced by antigen-presenting cells regulates the proliferation and activation of intestinal epithelial cells through STAT transcription factors [51]. We found that E7, D1, and Q13 can regulate pro-inflammatory and anti-inflammatory cytokine production to varying degrees, and E7 treatment exhibited the highest effect.

This study provides the first evidence that E7 exhibits excellent antioxidant activity and can improve UC in mice, which is expected to inspire more researchers to explore the probiotic properties of *Limosilactobacillus fermentum*. However, it is still necessary to determine the specific mechanisms by which E7 improves UC in mice through relevant signaling pathways. Future research will also require the use of metagenomic analysis to further elucidate the relevant genes in the gut that can mitigate inflammatory responses. Moreover, the other potential functions of E7 require further exploration. More research and functional evaluation on different strains of *Limosilactobacillus fermentum* are needed, which could potentially accelerate their development.

## 5. Conclusions

**Figure 8:**
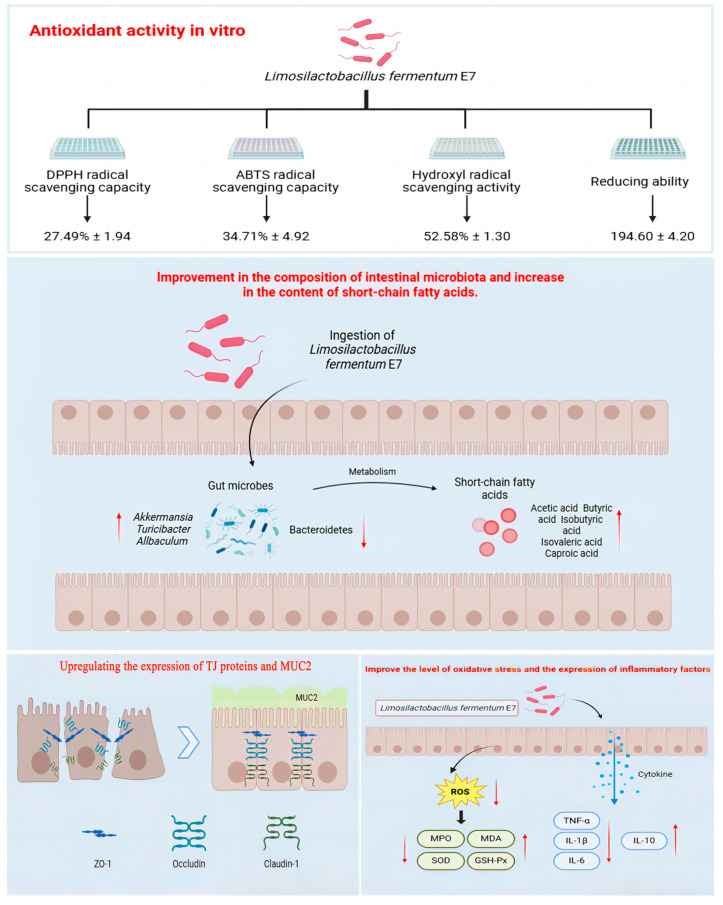
Diagram illustrating the mechanism by which *Limosilactobacillus fermentum* E7 relieves UC (created in BioRender). The red up arrow indicates an increase in expression, and the down arrow indicates a decrease in expression. The blue downward arrow indicates the delivery direction.

We identified five probiotics with good antioxidant effects through in vitro antioxidant screening and used them in an in vivo model of UC in mice. Physicochemical indices and pathological section results showed that E7, D1, and Q13 all induced significantly improvement in IBD symptoms in the mice. Therefore, downstream analysis was conducted on these three probiotics for the gut microbiota, metabolites, and cytokines. The results revealed that compared with D1 and Q13, E7 was more effective in alleviating UC. The specific mechanism of action is shown in Figure 8. In a word, we have proved the feasibility of E7 in improving UC in mice, and this discovery may provide a promising method for realizing safer and more effective enhancement strategies for UC.

## Figures and Tables

**Table 1:** Screening of antioxidant probiotics in vitro.

Strain Name	Strain Code	DPPH Radical Scavenging %	ABTS Radical Scavenging %	Hydroxyl Radical Scavenging %	Reducing Ability
*Enterococcus durans*	DS1	—	15.2 ± 2.71	28.01 ± 3.03	86.33 ± 1.13
*Enterococcus durans*	DS4	21.78 ± 1.30	22.73 ± 3.51	14.81 ± 1.65	80.83 ± 3.58
*Enterococcus lactis*	DS2b	10.50 ± 1.17	21.42 ± 1.75	29.06 ± 1.62	84.33 ± 1.93
*Pediococcus acidilactici*	DS6	25.81 ± 1.46	20.33 ± 4.41	39.67 ± 1.19	85.8 ± 3.08
*Pediococcus acidilactici*	P21	12.11 ± 2.40	21.40 ± 1.04	30.90 ± 1.10	77.70 ± 5.27
*Lactiplantibacillus plantarum*	H6	33.44 ± 1.50	37.81 ± 3.12	55.36 ± 1.35	188.60 ± 1.43
*Lactiplantibacillus plantarum*	H8	-	25.59 ± 2.59	27.17 ± 4.09	79.23 ± 0.79
*Lactiplantibacillus plantarum*	P3	13.70 ± 1.58	17.83 ± 4.37	40.01 ± 5.61	85.56 ± 1.96
*Lactiplantibacillus plantarum*	P4	14.80 ± 0.96	18.72 ± 1.67	32.95 ± 1.47	127.9 ± 3.30
*Lactiplantibacillus plantarum*	P19	12.65 ± 2.59	26.74 ± 2.63	28.69 ± 2.81	91.00 ± 2.60
*Lactiplantibacillus plantarum*	P20	—	17.64 ± 3.56	29.24 ± 2.71	81.36 ± 4.64
*Lactiplantibacillus plantarum*	T3	13.87 ± 0.90	17.82 ± 2.58	13,60 ± 0.70	83.63 ± 2.86
*Lactiplantibacillus plantarum*	K8	—	7.62 ± 2.78	29.68 ± 4.85	86.90 ± 1.64
*Latilactobacillus sakei*	LC4	22.61 ± 3.13	12.72 ± 3.63	46.15 ± 1.60	125.97 ± 3.10
*Latilactobacillus sakei*	QC9	31.85 ± 2.30	36.61 ± 1.31	48.94 ± 3.15	155.43 ± 1.04
*Limosilactobacillus fermentum*	F6	17.92 ± 1.77	24.26 ± 5.61	43.46 ± 2.74	98.60 ± 2.26
*Limosilactobacillus fermentum*	E7	27.49 ± 1.94	34.71 ± 4.92	52.58 ± 1.30	194.60 ± 4.20
*Limosilactobacillus fermentum*	T20	22.16 ± 5.52	32.51 ± 2.52	—	97.3 ± 3.63
*Bacillus subtills*	D1	37.63 ± 2.22	41.88 ± 1.26	60.15 ± 2.44	283.10 ± 3.81
*Bacillus subtilis* subsp. *subtilis str*.	YS1	24.09 ± 3.35	24.90 ± 3.50	50.30 ± 4.97	286.9 ± 1.27
*Bacillus subtilis* subsp. *subtilis str*.	YK6	25.50 ± 1.78	28.63 ± 3.62	48.17 ± 1.73	110.7 ± 2.32
*Bacillus licheniformis*	Q13	37.64 ± 1.67	42.49 ± 0.93	57.00 ± 2.16	289.80 ± 3.48
*Bacillus paralicheniformis*	Q23	28.43 ± 1.31	38.37 ± 2.72	59.63 ± 3.36	234.4 ± 1.72
*Bacillus amyloliquefaciens*	Q25	29.67 ± 1.28	39.71 ± 2.94	43.43 ± 2.67	139.3 ± 4.43
*Bacillus amyloliquefaciens*	Q221	28.46 ± 4.27	30.84 ± 1.72	42.94 ± 1.36	106.7 ± 1.17
*Bacillus stercoris*	YJ2	26.68 ± 1.30	27.85 ± 1.83	32.10 ± 1.61	226.00 ± 2.31
*Bacillus safensis*	YB1	25.67 ± 1.51	36.39 ± 4.36	49.36 ± 1.44	106.2 ± 1.12
*Weissella confusa*	M1	14.39 ± 1.73	15.73 ± 0.69	12.23 ± 0.90	193.40 ± 3.29
*Leuconostoc mesenteroides*	M2	16.90 ± 3.88	17.31 ± 1.73	33.56 ± 2.73	89.74 ± 3.64
*Lactobacillus brevis*	S10	10.60 ± 1.33	10.60 ± 1.52	17.80 ± 2.00	94.00 ± 2.90

## Data Availability

The original contributions presented in the study are included in the article/Supplementary Material. Further inquiries can be directed to the corresponding author.

## Supplementary Materials

The following supporting information can be downloaded at: https://www.mdpi.com/article/10.3390/foods14010137/s1, Table S1. The name of the probiotic strain and its source. Table S2. Calculated DAI score. Table S3. Primer sequences used for qRT-PCR.
