# Supplementary material for: Uncovering the Beneficial Role of Limosilactobacillus fermentum E7 Exhibiting Antioxidant Activity in Ameliorating DSS-Induced Ulcerative Colitis in a Murine Model
Source: Foods. 2025 Jan 6;14(1):137. doi: 10.3390/foods14010137 (PMC11719463; doi:10.3390/foods14010137)
Supplement: Supplementary file 1 [file foods-14-00137-s001.zip › foods-3325597-SM.pdf]

Table S1 The name of the probiotic strain and its source.

Strain code	Strain name	Strain source	Strain code	Strain name	Strain source
DS1	*Enterococcus durans*	Rice cake	F6	*Limosilactobacillus fermentum*	Kimchi
DS4	*Enterococcus durans*	Rice cake	E7	*Limosilactobacillus fermentum*	Kimchi
DS2b	*Enterococcus lactis*	Rice cake	T20	*Limosilactobacillus fermentum*	Tang zi mian
DS6	*Pediococcus acidilactici*	Rice cake	D1	*Bacillus subtills*	Soycheese
P21	*Pediococcus acidilactici*	Pickled pepper	YS1	*Bacillus subtilis subsp. subtilis str.*	Su zi ye salted vegetable
H6	*Lactiplantibacillus plantarum*	Sticky dough	YK6	*Bacillus subtilis subsp. subtilis str.*	Pickled radish
H8	*Lactiplantibacillus plantarum*	Sticky dough	Q13	*Bacillus licheniformis*	Chung
P3	*Lactiplantibacillus plantarum*	Pickled pepper	Q23	*Bacillus paralicheniformis*	Chung
P4	*Lactiplantibacillus plantarum*	Pickled pepper	Q25	*Bacillus amyloliquefaciens*	Chung
P19	*Lactiplantibacillus plantarum*	Pickled pepper	Q221	*Bacillus amyloliquefaciens*	Chung
P20	*Lactiplantibacillus plantarum*	Pickled pepper	YB1	*Bacillus safensis*	Babao cai salted Cabbage
T3	*Lactiplantibacillus plantarum*	Tang zi mian	YJ2	*Bacillus stercoris*	Mustard silk
K8	*Lactiplantibacillus plantarum*	Rutabaga	M1	*Weissella confusa*	Soycheese
LC4	*Latilactobacillus sakei*	Sausage	M2	*Leuconostoc mesenteroides*	Rice cake
QC9	*Latilactobacillus sakei*	Sausage	S10	*Lactobacillus brevis*	Su zi ye salted vegetable

Table S2 Calculated DAI score.

Score	Weight loss (%)	Food intake decrease(g)	Fecal consistency and occult blood
0	0-5	0-0.5	normal faeces
1	5-10	0.5-1	loose stool

2	10-15	1-1.5	watery diarrhea
3	15-20	1.5-2	diarrhea with blood
4	over 20	over 2	watery diarrhea, with a lot of blood.

Table S3 Primer sequences used for qRT-PCR.

Genes		Primer sequences (5'-3')
GAPDH	Forward	5'-GGTTGTCTCCTGCGACTTCA-3'
	Reverse	5'-TGGTCCAGGGTTTCTTACTCC-3'
IL-1β	Forward	5'-GGGAAACAACAGTGGTCAGG-3'
	Reverse	5'-CCATCAGAGGCAAGGAGGA-3'
IL-6	Forward	5'-TCTTGGGACTGATGCTGGTG-3'
	Reverse	5'-CATGTGTAATTAAGCCTCCGACT-3'
IL-10	Forward	5'-TTGCCAAGCCTTATCGGA-3'
	Reverse	5'-ACCCAGGGAATTCAAATGC-3'
TNF-α	Forward	5'-CTGTGAAGGGAATGGGTGTT-3'
	Reverse	5'-GTCACTGTCCCAGCATCTTGT-3'

GAPDH, Glyceraldehyde 3-phosphate dehydrogenase; IL-1β, Interleukin 1β; IL-6, Interleukin 6; IL-10, Interleukin 10; TNF-α, Tumor Necrosis Factor-alpha.
